# The dinucleotide CG as a genomic signalling module

**DOI:** 10.1186/1868-7083-5-S1-S1

**Published:** 2013-08-19

**Authors:** Adrian Bird

**Affiliations:** 1 The Wellcome Trust Centre for Cell Biology, University of Edinburgh, Edinburgh, EH9 3JR, UK

## 

The DNA sequence 5’CG (CpG) is self-complementary and can exist in three major chemical forms depending on the modification status of its cytosine moiety. To understand the functional significance of the CpG dinucleotide, we study proteins that bind either its methylated or unmethylated form. These proteins are likely mediators of CpG signalling that influence chromatin modification and thereby genome activity. The local density of CpG varies dramatically within genomic DNA. In the bulk genome CpG is rare and highly methylated, but in so-called “CpG islands” (CGIs) it is dense and usually non-methylated. A signature histone mark at non-methylated CGIs and also at transcriptionally active genes is trimethylation of histone H3 lysine 4. We are exploring the mechanisms by which DNA sequence features that are shared by all CGIs influence this and other epigenetic marks. In contrast, proteins that interact with methyl-CpG are thought to promote gene silencing by recruiting transcriptional corepressors. In particular mutations in the gene for the methyl-CpG binding protein MeCP2 cause the autism spectrum disorder Rett Syndrome. By studying MeCP2 we are learning about its partner proteins that mediate effects on chromatin. These findings allow us to evaluate competing models for MeCP2 function and they illuminate the biology of DNA methylation and the molecular basis of this neurological condition.

